# Joint action goals reduce visuomotor interference effects from a partner’s incongruent actions

**DOI:** 10.1038/s41598-019-52124-6

**Published:** 2019-10-28

**Authors:** Sam Clarke, Luke McEllin, Anna Francová, Marcell Székely, Stephen A. Butterfill, John Michael

**Affiliations:** 1 Department of Philosophy, York University, S900 Ross Building, 4700 Keele Street, Toronto, Ontario M3J 1P3 Canada; 2 Department of Cognitive Science, Central European University, Október 6. u. 7, Budapest, 1051 Hungary; 3 Department of Philosophy, The University of Warwick, University of Warwick, Coventry, CV4 7AL UK

**Keywords:** Motor control, Human behaviour

## Abstract

Joint actions often require agents to track others’ actions while planning and executing physically incongruent actions of their own. Previous research has indicated that this can lead to visuomotor interference effects when it occurs outside of joint action. How is this avoided or overcome in joint actions? We hypothesized that when joint action partners represent their actions as interrelated components of a plan to bring about a joint action goal, each partner’s movements need not be represented in relation to distinct, incongruent proximal goals. Instead they can be represented in relation to a single proximal goal – especially if the movements are, or appear to be, mechanically linked to a more distal joint action goal. To test this, we implemented a paradigm in which participants produced finger movements that were either congruent or incongruent with those of a virtual partner, and either with or without a joint action goal (the joint flipping of a switch, which turned on two light bulbs). Our findings provide partial support for the hypothesis that visuomotor interference effects can be reduced when two physically incongruent actions are represented as mechanically interdependent contributions to a joint action goal.

## Introduction

From handshakes to music-making, dance and team sports, social interactions often require an efficient means of tracking others’ actions while simultaneously planning and executing actions of one’s own^1^. A basketball player, for example, must monitor and anticipate her teammate’s movements in order to successfully contribute to a pick and roll play.

Given the broad range of social interactions in which it is important to anticipate, monitor and respond to others’ actions, it is no surprise that a considerable amount of research has been devoted to investigating how we achieve this^2–5^. An influential idea that has emerged is that the representation of others’ actions is often supported by one’s own motor system, implying that representations of others’ actions are often functionally equivalent to the representations involved in action production^2–4,6,7^. As a result, the observation of others’ actions can result in action representations that do not clearly distinguish self from other^8–10^.

An upshot is that the observation of others’ actions can give rise to representations that interfere with one’s own task performance. In a striking illustration of this, Brass *et al*.^2^ found that participants who were instructed to produce finger movements in response to symbolic cues responded more quickly when simultaneously observing irrelevant finger movements that were physically congruent to the ones they were instructed to produce, and more slowly when simultaneously observing irrelevant finger movements that were physically incongruent to these. These findings – and others that build on them^11–13^ – are taken to indicate that, when observing others’ actions, we automatically represent those actions using motor representations of the same type as those subserving action production.

This neatly explains why the observation of congruent actions facilitates task performance, while the observation of incongruent actions leads to visuomotor interference effects. However, it also raises a challenge. This is because many joint actions require individuals to produce *physically incongruent* yet *complementary* actions^14^. A proficient basketball player, for example, may need to coordinate her movement towards the basket with her teammate’s passing of the ball. But if tracking her teammate’s action elicits motor representations that compete with those underpinning the action she herself must perform, then they may interfere with her own action preparation. In more general terms: where the tracking of others’ actions involves motor representations that are functionally equivalent to the representations underpinning action production, this could give rise to interference effects and prove counter-productive in many cases of joint action.

This problem can, however, be overcome. In a recent paper by Sacheli, Arcangeli, & Paulesu^15^ participants played learned melodies *with*, or merely *alongside*, a virtual partner. In both cases, this required them to sequentially produce actions that were either physically congruent (e.g. point-point) or physically incongruent to those that had just been produced by the partner (e.g. point-grasp). When participants and their partners performed these actions alongside one another (i.e. in a Non-Interactive Condition) performance was affected by the physical (in)congruence of the movements, as expected. But, when these actions were directed towards a joint action goal (i.e. the joint production of a single melody in a Joint Action Condition), physical congruence became irrelevant: task performance was affected by a reversal in movement-note associations, but not by the congruence or incongruence of the two agents’ movements. This raises the question: why would doing something in the context of a joint action eliminate interference from the perception of incongruent movements but create interference from the perception of anomalous sounds?

Sacheli *et al*.’s proposed answer is that the representation of a joint action goal enables joint action partners to integrate representations of their own and their partner’s actions within a single *dyadic* (multi-person) motor plan^15^. As they put it, this dyadic motor plan enables agent’s to select appropriate responses to their partner’s actions on the basis of their predicted outcomes (e.g. the production of a musical note). This explains why anomalous movement-note associations would have generated interference in their study. However, it does not appear to explain why the joint action frame would have reduced interference from physically incongruent movements. In principle, integrating representations of incongruent movements within a larger motor plan could have increased interference effects instead^16^.

**Figure 1:**
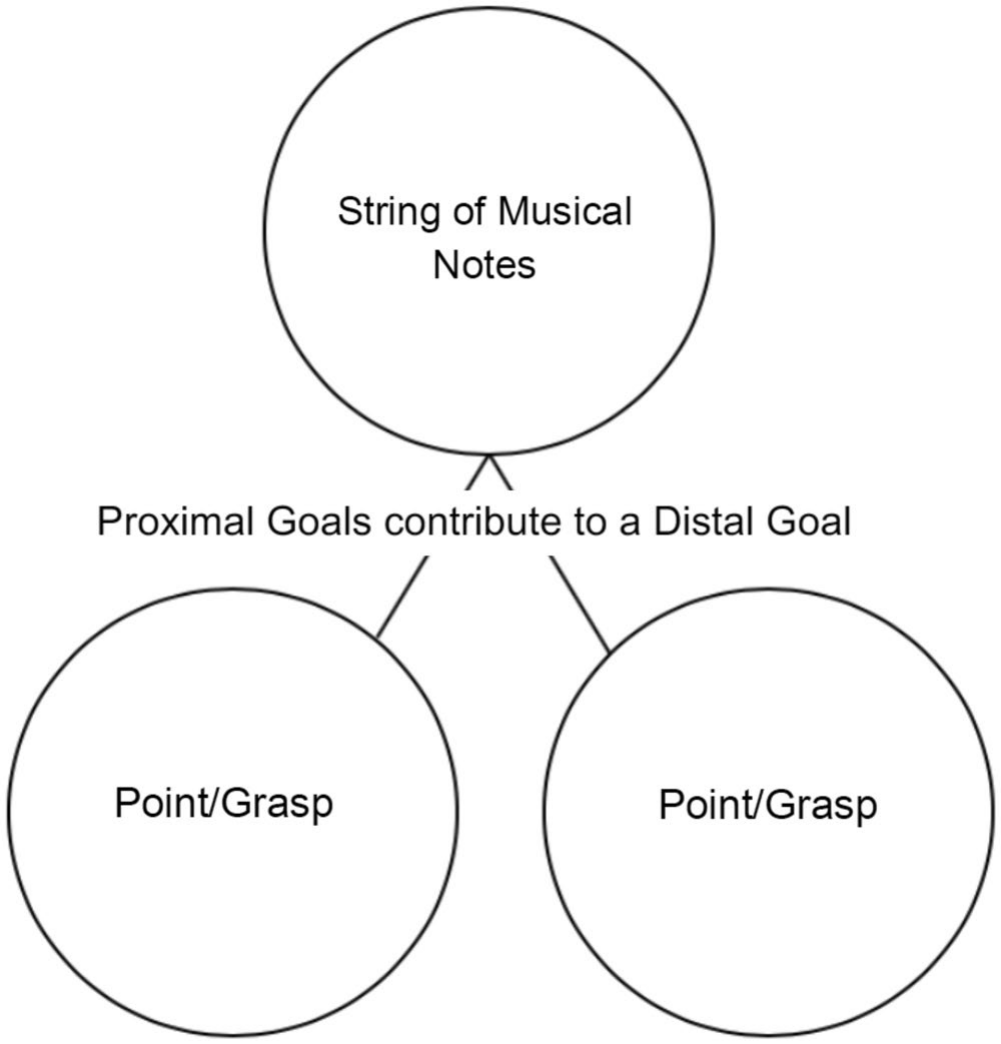
Two physically incongruent actions become part of a larger Joint Action plan. If there is no need to represent the other partner’s *incongruent* action (i.e. if the agent can produce their contribution to the joint action without taking their partner’s behaviour into account), then this may allow agents to bypass the representation of a partner’s actions altogether, allowing interference effects to be reduced or avoided. However, when one agent has to select an action based on which action their partner performs, their individual action cannot be represented only in terms of the more distal joint action or its goal.

One possibility, left open by the aforementioned study, is that a joint action frame may lead participants to represent their partner’s actions in relation to a more distal joint action goal (i.e. a string of musical notes) instead of the more proximal goals that bring this about (i.e. grasping or pointing). In cases where the physical incongruence of the actions only obtains at the level of these more proximal goals, this might allow agents to bypass the representation of their partners’ physically incongruent movements altogether, reducing or eliminating visuomotor interference effects (See Fig. 1). The trouble is: there seem to be cases of joint action where it is not sufficient to bypass the representation of a partner’s proximal goal altogether and to merely consider the more distal outcome of the joint action goal. Rather, as illustrated by the basketball players mentioned above, it is often necessary to represent the more proximal goals of a partner’s action in order to select actions that would complement these with respect to the more distal joint action goal. Indeed, this can be true of even the most basic motor movements involved. Thus, basic questions remain. Specifically: can the introduction of a distal joint action goal reduce visuomotor interference effects in cases where incongruent proximal goals are contingently related to one another, and attention to these is required for the selection of appropriate motor movements? And, if so, how might this be achieved?

In addressing the latter question, a natural starting point is the observation that action production typically involves the simultaneous representation of multiple, instrumentally related actions at multiple, instrumentally related levels of abstraction^17–20^. For example, we represent the action of turning the steering wheel not only at the level of the comparably distal goal (turned steering wheel) but also at the level of comparatively proximal goals, designed to bring this about (e.g. raised left arm; lowered right arm). Importantly, this hierarchical structure must capture instrumental relations between these different goals. Plainly, proximal goals must function to bring about comparatively distal goals. But, in addition to this, the comparatively proximal goals must (themselves) be sensitive to each other such that a modification to one will lead others to change appropriately. For instance, one need not bother moving one’s arms if one is no longer grasping the wheel; and even when one is grasping the wheel, it may be no use raising one’s left arm if one does not simultaneously lower one’s right arm.

Here, the individual agent must simultaneously produce physically incongruent movements (arm lifting and arm lowering). But, in this case, it is not possible to avoid motoric interference by simply considering each arm’s movement independently of the other, or by simply considering the more distal goal outcome to which these are both directed (a turned wheel). This is because all of these goals are interrelated. Thus, the introduction of the more distal goal must change the way in which the more proximal goals are represented. Specifically, it must lead to their representation as interrelated, and not simply independent contributions to a larger action.

**Figure 2:**
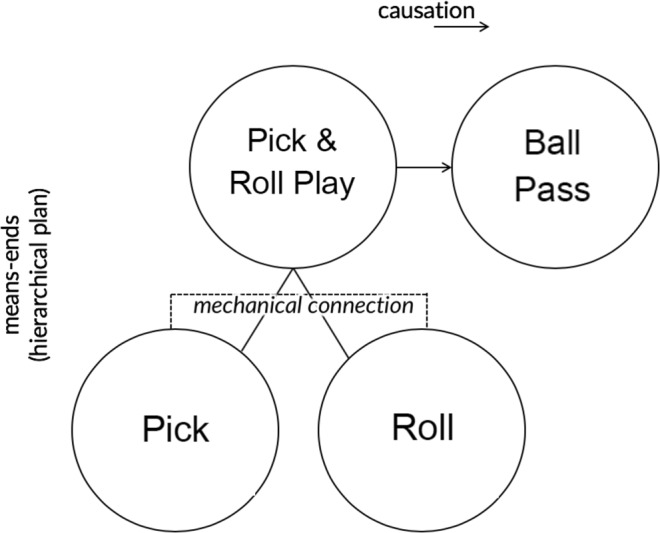
Where one agent has to select an action based on which action the other performs, interference effects may be reduced if the agent can represent both actions as interrelated components of a single goal and not only in terms of the more distal goal (e.g. passing the ball, in a pick and roll play).

This raises the possibility that the actions of our joint action partners can be represented in relation to the same action hierarchy (See Fig. 2). Here, the introduction of a comparably distal joint action goal might enable the physically incongruent movements of self and other to be represented as interrelated components of a plan to bring about the joint action goal. If this is possible, then it might reduce or even eliminate interference from the observation of a partner’s physically incongruent movements, even when success in joint action requires one’s selective response to these. Thus, we hypothesise that where agents represent their actions as interrelated components of a plan to bring about a joint action goal, each partner’s movements need not always be represented in relation to distinct, incongruent proximal goals. Instead, they might be represented as interrelated contributions to a single goal. If true, the joint action frame could potentially reduce or even eliminate visuomotor interference effects arising from the observation of what an outsider might take to be a physically incongruent action.

**Figure 3:**
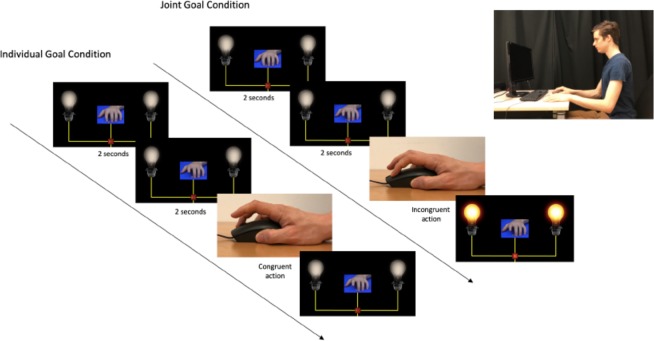
Illustration of the task. Participants were instructed to lift the same finger as the hand in the video when a ‘1’ is displayed (Congruent Condition) and to lift the other finger when a ‘2’ is displayed (Incongruent Condition). The left side illustrates the Individual Goal Condition, in which the lights never turn on. The right side illustrates the Joint Action Goal Condition, in which the lights are turned on when two conditions are fulfilled: the number cue (‘2’) indicates that the participant should perform the ‘incongruent’ action, and the participant correctly does so.

To test this, we adapted Brass and colleagues’^12^ paradigm to incorporate a joint action goal, namely turning on two light bulbs by jointly flicking a switch. Here, participants were required to perform one of two finger-lifting movements depending on which numerical cue was presented on a screen, in between a virtual partner’s index and middle fingers (See Fig. 3). These movements could be physically congruent or physically incongruent with a movement performed by the virtual partner. In a Joint Action Goal Condition, lightbulbs were turned on when the participant and the partner simultaneously performed physically incongruent actions, but not when they performed physically congruent actions (something about which our hypothesis makes no predictions). In the Individual Goal Condition, the lights were never turned on (i.e. there was no joint action goal). We reasoned that if participants are able to utilize the joint action goal (turning on the lightbulbs) to represent a planning structure in which their partner’s movement forms a complementary and mutually interrelated contribution, then the physical incongruence of their own and the partner’s movement should be less relevant. This generates the prediction that we should observe reduced visuomotor interference effects in the Joint Action Goal Condition compared to the Individual Goal Condition. In other words, the difference in response times between Congruent trials (wherein the participant and the partner lift the same fingers) and Incongruent trials (wherein the participant and the partner lift different fingers) should be smaller in the Joint Action Goal Conditions than in the Individual Goal Condition.

The predictions, sample size, methods, and planned analyses were all pre-registered before data collection and can be accessed at: http://aspredicted.org/blind.php?x=cr4cg2. Unless otherwise noted, we implemented all steps as pre-registered.

## Results

To control for speed-accuracy tradeoffs, reaction time (RT) for correct responses and hit rates (HR) were merged into inverse efficiency scores (IES), a combined measure which homogenizes different patterns of speed-accuracy trade-offs (IES)^21^, by dividing RTs by accuracy for each condition in each group (lower scores mean more efficient responses). We also analyzed the participants’ RT’s.

**Figure 4:**
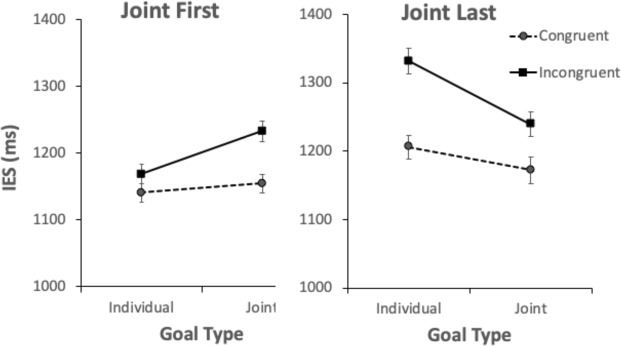
2 × 2 × 2 ANOVA. Mean Inverse Efficiency Scores (IES) are plotted separately for Congruent and Incongruent trials in the Joint Action Goal and the Individual Goal Conditions, for each group. Error bars represent the within-subject confidence intervals^29,30^.

For the IES, we conducted a 2 × 2 × 2 mixed ANOVA with Jointness (Joint vs Individual Action Goal) and Congruence (Congruent vs Incongruent) as within participants factors, and Group (Joint First, Joint Last) as a between participants factor. The ANOVA revealed a significant main effect of Congruence *F*(1,70) = 44.41, *p* < 0.001, ηp2 = 0.39, with lower IES in the Congruent condition, (*M* = 1168.67, *SD* = 211.75) than in the Incongruent condition (*M* = 1243.39, *SD* = 230.94); but no significant main effect of Jointness, *F*(1,70) = 0.49, *p* = 0.48, ηp2 = 0.007, and no significant main effect of Group *F*(1,70) = 1.72, *p* = 0.19, ηp2 = 0.02. There was no significant interaction between Jointness and Congruence, *F* (1,70) = 0.05 *p* = 0.82, ηp2 = 0.001, no significant interaction between Congruence and Group, *F*(1,70) = 3.68, *p* = 0.06, ηp2 = 0.05 (although this was close to significance, we cannot make conclusions on the basis of this statistic), but a significant interaction between Jointness and Group, *F*(1,70) = 9.61, *p* = 0.003, ηp2 = 0.12. There was also a three way interaction between Jointness, Group and Congruence, *F*(1,70) = 14.49, *p* < 0.001, ηp2 = 0.17 (see Fig. 4).

Post-hoc t-tests for the Joint First Group revealed IES did not differ between congruent and incongruent trials for the Individual Goal condition, *t*(35) = −1.99, *p* = 0.054, *d* = −0.33 but IES were significantly lower for congruent trials than incongruent trials for the Joint Goal condition, *t*(35) = −4.89, *p* < 0.001, *d* = −0.82. Congruent trials’ IES in the Individual Goal condition were not significantly different from congruent trials’ IES in the Joint Goal condition *t*(35) = −0.55, *p* = 0.59, *d* = −0.09 and incongruent trials’ IES in the Individual Goal condition were not significantly different from incongruent trials’ IES in the Joint Goal condition, *t*(35) = −1.93, *p* = 0.06, *d* = −0.32.

Post-hoc t-tests for the Joint Last Group revealed that congruent IES were significantly lower than incongruent trials’ IES for the Individual Goal condition, *t*(35) = −5.25, *p* < 0.001, *d* = −0.88 and congruent trials’ IES were significantly lower than incongruent trials’ IES for the Joint Goal condition, *t*(35) = −3.43, *p* = 0.002, *d* = −0.57. Congruent trials’ IES in the Joint Goal condition were not different from congruent trials’ IES in the Individual Goal condition, *t*(35) = 2.05, *p* = 0.05, *d* = 0.34, however incongruent trials’ IES in the Joint Goal condition were significantly lower than incongruent trials’ IES in the Individual Goal condition, *t*(35) = 3.98, *p* < 0.001, *d* = 0.66.

**Figure 5:**
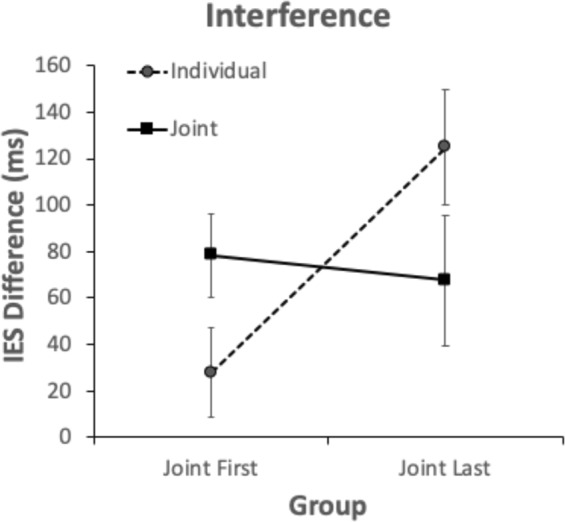
2 × 2 ANOVA. Mean difference between Congruent and Incongruent Inverse Efficiency Scores (IES) for Joint Action Goal and the Individual Goal Conditions, for each group. Error bars represent the within-subject confidence intervals^29,30^.

We believe that the three-way interaction was likely the result of the incongruent trials causing comparatively little visuomotor interference in the first block of trials when there was a Joint Action Goal, compared to when there was not a Joint Action Goal. To further investigate the three-way interaction, we subtracted the IES of congruent trials from the IES of incongruent trials (IES difference) for each participant, for each condition, giving us an index of how much the incongruent trials interfered with participants’ responses in each of the conditions. We conducted a 2 × 2 mixed ANOVA, with Jointness as a within participants factor, and Group as a between participants factor, which revealed no main effect of Jointness, *F*(1,70) = 0.05, *p* = 0.82, ηp2 = 0.001, and no main effect of Group, *F*(1,70) = 3.69 *p* = 0.06, ηp2 = 0.05 (although this is close to significance, it does not permit us to draw any conclusions). However, there was an interaction between Jointness and Group, *F*(1,70) = 14.49, *p* < 0.001, ηp2 = 0.17 (see Fig. 5). Bonferroni corrected post-hoc t-tests revealed that Joint Action Goal IES differences were significantly larger than Individual Goal IES differences for the Joint First group, *t*(35) = −2.64, *p* = 0.012, *d* = −0.44, and Joint Action Goal IES differences were significantly smaller than Individual Goal IES differences for the Joint Last Group, *t*(35) = 2.74, *p* = 0.01, *d* = 0.46. There was no significant difference between the Joint First group and Joint Last group for Joint Action Goal Trials, *t*(70) = 0.43, *p* = 0.67, *d* = 0.1, but Joint First IES differences were significantly smaller than Joint Last IES differences for Individual Goal trials, *t*(70) = −3.51, *p* < 0.001, *d* = 0.83.

## Discussion

Our results revealed a three-way interaction of Congruence, Jointness and Group. In the group which performed the Joint Action Goal Condition last, the difference between congruent and incongruent trials was significantly smaller when there was a joint action goal than when there was none. In the group which performed the Joint Action Goal Condition first, in contrast, the difference between congruent and incongruent trials was significantly greater when there was a joint action goal than when there was none.

The results from the group which performed the Joint Action Goal Condition last indicate that a joint action goal representation may, as we predicted, reduce visuomotor interference effects arising from the observation of a physically incongruent action. These results build upon earlier research indicating that the visuomotor interference effects arising from the perception of a virtual partner’s physically incongruent movement can be reduced when two physically incongruent movements are represented as complementary contributions to a joint action goal^15,22^. Our results extend this research by suggesting that a joint action goal representation can reduce visuomotor interference effects even when agents must detect a joint action partner’s physically incongruent movement while simultaneously producing actions that are contingently related to these. This supports the hypothesis that where agents represent their actions as interrelated components of a plan to bring about a joint action goal, the movements of each partner’s effectors need not always be represented in relation to distinct, incongruent proximal goals. Instead they can be represented as interrelated contributions to a single goal – especially if the movements are, or appear to be, mechanically linked to a more distal joint action goal.

The results from the group which performed the Joint Action Goal Condition first, in contrast, are not consistent with our prediction. For this group, the joint action framing did not lead to a reduction of visuomotor interference effects. Indeed, the difference in performance between congruent and incongruent trials was significantly larger in the Joint Action Goal condition than in the Individual Goal condition for this group. This pattern may be partially be attributed to a carryover effect: having completed the Joint Action Goal Condition first, participants in this group may have continued representing incongruent trials as contributing towards a joint action goal, even once this goal (turning on lightbulbs) had been removed. This conjecture would explain why, for participants in this group, performance on incongruent trials did not worsen from the first block (Joint Action Goal Condition) to the second block (Individual Goal Condition). However, it does not explain why performance on congruent trials did worsen slightly from the first block (Joint Action Goal Condition) to the second block (Individual Goal Condition). We might speculate that this was due to fatigue: it may have been difficult to maintain the high level of performance that we observed in this group on congruent trials in the first block (Joint Action Goal Condition).

The mixed pattern of results between our two groups underscores the broader point that we should not expect the introduction of a joint action goal representation to always eliminate or reduce visuomotor interference effects arising from the concurrent performance and perception of physically incongruent actions. And indeed, there is evidence that it does not always do so. For instance, it has been shown that under certain circumstances joint action goal representations can lead to an increase in this form of interference. In one study^16^, participants were instructed to draw either circles or straight vertical lines on tablet screens resting on a table in front of them while a second participant, sitting diagonally across from them, performed the other (i.e. incongruent) action. On a screen directly in front of each participant, they could see the outcome of their partner’s action (i.e. a straight line or a circle appearing on the screen). One group of participants (the Joint Action Condition) were informed that, together, their own and their partner’s drawings constituted complementary components of a single drawing. A second group of participants (the Parallel Condition) were informed that the other agent’s action was irrelevant to their own task. Within this setup, the joint action goal (i.e. in the Joint Action condition) led to an increase in interference effects. If our hypothesis is correct, this finding is not surprising: since participants did not have to identify their partner’s movement in order to select their own movement, and since they were unable to see the outcome of their combined efforts, they did not perceive their own and their partner’s actions as mechanically linked to a more distal joint action goal. Thus, participants simply represented their own and their partner’s movements in relation to distinct and incongruent proximal goals.

This would be consistent with research showing the facilitatory effects of Lissajous plots on bimanual coordination^23,24^. In this research, participants are instructed to perform two separate rhythmic actions, one with each hand. Lissajous plots are used to display the location of one limb on the x-axis, and the location of the other limb on the y-axis, as well as the location of a dot integrating the locations of the two hands. This visual feedback enables participants to represent the two hand movements as mechanically linked to a single, combined outcome. As a result, they are able to maintain otherwise unstable phase relations^25,26^.

It would be important for future research to investigate other contexts in which individuals must efficiently represent and respond to others’ physically incongruent actions. In particular, it would be valuable to probe competitive scenarios in which there are no joint action goals but in which the outcomes of two agents’ are interdependent and to investigate the extent to which the representation of a partner’s actions can be modulated by the degree of coordination required by a joint action goal. Future research should also investigate the neural mechanisms that underpin the integration of physically incongruent actions into unified motor plans. One important starting point in this respect is provided Sacheli, Tieri, Aglioti, & Candidi’s^22^ study demonstrating that virtual lesions (created using continuous theta-burst stimulation) in the left anterior intraparietal sulcus led to an increase in visuomotor interference effects in a scenario in which participants observed a partner’s action and were required to select a physically incongruent action to perform synchronously.

## Methods

### Participants

Using G∗Power 3.1^27^ we determined that a sample size of thirty would provide 80% statistical power for detecting a small-to-medium-sized effect of the interaction of the two main factors, Jointness (Joint Action Goal vs Individual Goal) and Congruence (Congruent vs Incongruent), assuming a two-way repeated measures ANOVA and an alpha level of 0.05. We therefore recruited 30 participants. Because of the high exclusion rate in this initial data collection, we had to recruit more participants to replace those who had been excluded, and overestimated the number of participants needed to compensate for exclusions. This resulted in a total of 36 participants. Due to experimenter error, all of these 36 participants were administered the Joint Last condition, so we then collected 36 participants for the Joint First group to counterbalance.

Thus, our sample included 72 participants (13 females; age range: 21–46, M = 27.1, SD = 4.77). All participants were recruited from student organizations in the Budapest and Warwick areas, were naïve to the purpose of the study, and reported normal or corrected to normal vision. All participants signed informed consent prior to the experiment, and received gift vouchers or money for their participation. The experiment was conducted in accordance with the Declaration of Helsinki and was approved by the (EPKEB) United Ethical Review Board for Research in Psychology.

### Apparatus and stimuli

The experiment was displayed on a 13-inch computer screen (resolution: 2560 × 1600 pixels, refresh rate: 60 Hz). The program for the experiment was written in OpenSesame Python^28^, with a frame rate of 17 frames per second. Figure 3 illustrates the task environment.

### Procedure

After giving their informed written consent, participants were seated alone at a desk in a lab room and provided with further instructions, after which they had the opportunity to ask clarificatory questions to the experimenter.

They then performed two test blocks (Joint Action Goal Condition and Individual Action Goal Condition) consisting of 80 trials each (40 Congruent, 40 Incongruent; 20 of each required index finger movements and 20 middle finger movements). Each test block was preceded by 4 practice trials (i.e. 8 in total). The order of test blocks was counterbalanced across participants; Incongruent and Congruent trials were evenly distributed across blocks and randomly mixed within each block.

At the beginning of each trial, participants were instructed to hold down the left and right buttons of the mouse with the index and middle fingers of their right hand. The stimuli were short video sequences of simple finger movements (lifting), as illustrated in Fig. 3. Then a picture of the hand was presented for 2000 ms with a number displayed between the index and middle finger, indicating the participant’s instruction for that trial: the participant was instructed to lift the same finger as her/his virtual partner on trials in which a ‘1’ was displayed below the picture of the hand (Congruent trials), and to lift the other finger on trials in which the number ‘2’ was displayed (Incongruent trials). Participants were instructed to respond as quickly and accurately as possible as soon as the virtual partner’s finger began to move. Next, a still frame of the virtual partner’s hand was displayed with the index or the middle finger having been lifted. The onset of this image was the participant’s go-signal to lift the appropriate finger.

In the Individual Action Goal Condition, the two light bulbs were displayed on the two sides of the screen (See Fig. 3) during all trials, but remained switched off at all times.

In the Joint Action Goal Condition, the instructions were the same as in the Individual Action Goal Condition except that participants were informed there would sometimes be a ‘bonus’ effect: when the participant and her/his partner correctly lifted different fingers (i.e. this was only possible on incongruent trials), they would jointly flip the switch, causing the lights to be switched on (See Fig. 3). Participants were explicitly informed that this could happen only on the trials in which the correct response was to perform the incongruent action.

At the end of each trial, the scene was displayed for 2000 ms. When the switch was flipped and the lightbulbs turned on, the scene was displayed with the switch having been flipped and the lightbulbs turned on. On trials when participants performed the incorrect action, the background turned red and the scene was displayed, otherwise unchanged, for 2000 ms. On trials when participants performed the correct action but the switch was not flipped and the lightbulbs not turned on, the scene was displayed just as in the previous frame.

### Data processing and analysis

For the analysis, we had several exclusion criteria. Firstly, we excluded four participants (three in the Joint First Group and one in the Joint Last Group) from all of our analysis as they had an unusually high rate of premature responses (all > 90%), meaning that it is likely that they did not understand the instructions and that their data cannot be relied on. Secondly, we excluded any participants with an overall accuracy more than 2.5 SD below the group mean (either Joint First group or Joint Last group) from all our analyses, as their data is likely unreliable. This resulted in the exclusion of 480 trials (7.6%) or 3 participants from the Joint First group, and 480 trials (7.6%) or 3 participants from the Joint Last group. Secondly, we excluded 72 (1.1%) premature responses (responses before the stimulus onset) from the Joint First group, and 58 (0.9%) premature responses from the Joint Last group, from all of our analysis. Thirdly, 147 (2.3%) trials with RTs more than 2.5 standard deviations (SDs) removed from the mean (calculated for each participant for each condition) were excluded from the Joint First group, and 128 trials (2.1%) were excluded from the Joint Last group. Finally, 240 trials (3.9%) incorrect responses for the Joint First group, and 297 trials (4.7%) from the Joint Last group were excluded from the RTs. Although these criteria were not pre-registered, we determined to apply them prior to analysing any data. Our rationale was that the hypothesis being tested pertained to the processes engaged when people perform actions while perceiving a physically incongruent action from a joint action partner; on trials on which participants committed errors, we could not be confident that these processes were actually engaged.

For each participant, we calculated the mean RT’s and accuracy (proportion correct), for congruent and incongruent trials for each condition (see Supplementary File for means per condition). We divided the RTs by the accuracy in order to compute Inverse Efficiency Scores (IES)^21^ as an index of efficiency, appropriately weighting speed and accuracy.

## Supplementary information

Appendix
