# Supplementary material for: Joint action goals reduce visuomotor interference effects from a partner’s incongruent actions
Source: Sci Rep. 2019 Oct 28;9:15414. doi: 10.1038/s41598-019-52124-6 (PMC6817939; doi:10.1038/s41598-019-52124-6)
Supplement: Supplementary file 1 — Appendix [file 41598_2019_52124_MOESM1_ESM.pdf]

Joint action goals reduce visuomotor interference effects from a partner's incongruent actions (Appendix/Supplementary Data)

Sam Clarke [a 1], Luke McEllin [b], Anna Francová [b], Marcell Székely [b], Stephen A. Butterfill [c], and John Michael [bc]

[a] Department of Philosophy, York University
S900 Ross Building, 4700 Keele Street, Toronto, Ontario, M3J 1P3, Canada

[b] Department of Cognitive Science, Central European University
Október 6. u. 7, Budapest 1051, Hungary

[c] Department of Philosophy, The University of Warwick
University of Warwick, Coventry, CV4 7AL, UK

RTs

Group	Trial	Congruence	Mean	SD	N
Joint First	Individual	Congruent	1092	179	36
		Incongruent	1128	171	36
	Joint	Congruent	1068	183	36
		Incongruent	1137	171	36
Joint Last	Individual	Congruent	1149	165	36
		Incongruent	1252	175	36
	Joint	Congruent	1133	155	36
		Incongruent	1176	134	36

Table 1: Mean and Standard Deviation of the reaction times for each of the experimental conditions

[1] spclarke@yorku.ca

Accuracy

Group	Trial	Compatibility	Mean	SD	N
Joint First	Individual	Congruent	.959	.034	36
		Incongruent	.964	.036	36
	Joint	Congruent	.936	.062	36
		Incongruent	.933	.068	36
Joint Last	Individual	Congruent	.955	.042	36
		Incongruent	.947	.055	36
	Joint	Congruent	.967	.036	36
		Incongruent	.950	.046	36

Table 2: Mean and Standard Deviation of the accuracy ratio (proportion of correct responses) for each of the experimental conditions
